# Diagnostic value and mechanism of plasma S100A1 protein in acute ischemic stroke: a prospective and observational study

**DOI:** 10.7717/peerj.14440

**Published:** 2023-01-10

**Authors:** Guo Hong, Tingting Li, Haina Zhao, Zhaohao Zeng, Jinglei Zhai, Xiaobo Li, Xiaoguang Luo

**Affiliations:** 1 Department of Neurology, Second Clinical Medical College of Jinan University, Shenzhen, China; 2 Department of Neurology, Yizheng People’s Hospital affiliated to Yangzhou University, Yangzhou, China; 3 Department of Neurology, Institutes of Brain Science, Jiangsu Subei People’s Hospital affiliated to Yangzhou University, Yangzhou, China; 4 School of Medicine, Southern University of Science and Technology, Shenzhen, China

**Keywords:** S100A1 calcium-binding protein, Nuclear transcription factor κB phosphorylation 65, Interleukin 6, Acute ischemic stroke, Cerebral infarction, Mechanism

## Abstract

**Background:**

Plasma S100A1 protein is a novel inflammatory biomarker associated with acute myocardial infarction and neurodegenerative disease’s pathophysiological mechanisms. This study aimed to determine the levels of this protein in patients with acute ischemic stroke early in the disease progression and to investigate its role in the pathogenesis of acute ischemic stroke.

**Methods:**

A total of 192 participants from hospital stroke centers were collected for the study. Clinically pertinent data were recorded. The volume of the cerebral infarction was calculated according to the Pullicino formula. Multivariate logistic regression analysis was used to select independent influences. ROC curve was used to analyze the diagnostic value of AIS and TIA. The correlation between S100A1, NF-*κ*B p65, and IL-6 levels and cerebral infarction volume was detected by Pearson correlation analysis.

**Results:**

There were statistically significant differences in S100A1, NF-*κ*B p65, and IL-6 among the AIS,TIA, and PE groups (S100A1, [230.96 ± 39.37] *vs* [185.85 ± 43.24] *vs* [181.47 ± 27.39], *P* < 0.001; NF-*κ*B p65, [3.99 ± 0.65] *vs* [3.58 ± 0.74] *vs* [3.51 ± 0.99], *P* = 0.001; IL-6, [13.32 ± 1.57] *vs* [11.61 ± 1.67] *vs* [11.42 ± 2.34], *P* < 0.001). Multivariate logistic regression analysis showed that S100A1 might be an independent predictive factor for the diagnosis of disease (*P* < 0.001). The AUC of S100A1 for diagnosis of AIS was 0.818 (*P* < 0.001, 95% CI [0.749–0.887], cut off 181.03, *J*max 0.578, *Se* 95.0%, *Sp* 62.7%). The AUC of S100A1 for diagnosis of TIA was 0.720 (*P* = 0.001, 95% CI [0.592–0.848], cut off 150.14, *J*max 0.442, *Se* 50.0%, *Sp* 94.2%). There were statistically significant differences in S100A1, NF-*κ*B p65, and IL-6 among the SCI,MCI, and LCI groups (S100A1, [223.98 ± 40.21] *vs* [225.42 ± 30.92] *vs* [254.25 ± 37.07], *P* = 0.001; NF-*κ*B p65, [3.88 ± 0.66] *vs* [3.85 ± 0.64] *vs* [4.41 ± 0.45], *P* < 0.001; IL-6, [13.27 ± 1.65] *vs* [12.77 ± 1.31] *vs* [14.00 ± 1.40], *P* = 0.007). Plasma S100A1, NF-*κ*B p65, and IL-6 were significantly different from cerebral infarction volume (S100A1, *r* = 0.259, *P* = 0.002; NF-*κ*B p65, *r* = 0.316, *P* < 0.001; IL-6, *r* = 0.177, *P* = 0.036). There was a positive correlation between plasma S100A1 and IL-6 with statistical significance (R = 0.353, *P* < 0.001). There was no significant positive correlation between plasma S100A1 and NF-*κ*B p65 (R < 0.3), but there was statistical significance (R = 0.290, *P* < 0.001). There was a positive correlation between IL-6 and NF-*κ*B p65 with statistical significance (R = 0.313, *P* < 0.001).

**Conclusion:**

S100A1 might have a better diagnostic efficacy for AIS and TIA. S100A1 was associated with infarct volume in AIS, and its level reflected the severity of acute cerebral infarction to a certain extent. There was a correlation between S100A1 and IL-6 and NF-*κ*B p65, and it was reasonable to speculate that this protein might mediate the inflammatory response through the NF-*κ*B pathway during the pathophysiology of AIS.

## Introduction

According to the latest global burden of disease data, acute ischemic stroke (AIS) was one of the main reasons for the increase in the number of years of life lost and all age disability-adjusted life years in China (Zhou et al., 2019). The prevalence and mortality of the population were as high as 1.6% and 1.1% (Wang et al., 2017). Acute ischemic stroke accounted for 69.6% of all types of stroke and was the leading cause of disability and death in rural and remote areas of China (Wang et al., 2017; Yang et al., 2013; Wang et al., 2015). In addition, there are approximately two million new cerebral infarcts annually, and medical treatment costs are in the tens of billions of dollars (Lopez et al., 2006). According to the American Heart Stroke Association Guidelines for the Early Management of Acute Ischemic Stroke and the AIS Endovascular Treatment Consensus (Sacks et al., 2018; Powers et al., 2018), the effective treatments for AIS mainly include intravenous thrombolysis and endovascular mechanical thrombectomy, but only for some patients within a specific time window. The vast majority of patients can only receive symptomatic treatment, and their prognosis is dismal, which is also the main reason for the high rates of recurrence, disability, and death among AIS patients. In the meantime, the diagnosis of early disease and the determination of its severity are difficult to assess. Multiple instability factors influence a diagnosis. This may also be related to the complex pathophysiological mechanisms of ASI. Therefore, searching for practical biological markers can help diagnose and determine the condition, especially for hospitals in remote areas or those lacking essential equipment.

Inflammation, abnormal coagulation, and atherosclerosis are the pathophysiological basis of ASI. The vital role of the immune-inflammatory response in all pathophysiological stages of AIS is now well recognized, both in the development of AIS and the prognosis of AIS. When an ischemic stroke occurs, the ischemic brain tissue activates glial cells that produce massive biochemical and inflammatory mediators, such as cytokines, chemokines, and pro-inflammatory enzymes (Bonaventura et al., 2016; Maida et al., 2020). S100A1 protein is a recently discovered novel inflammatory marker; it is also a member of the S100 protein family. It can be expressed in endothelial cells and binds to target proteins inside and outside the cell to play a role in signalling and neurotransmitter transmission, as well as being involved in the synthesis of certain enzymes (Gonzalez, Garrie & Turner, 2020; Baudier, Deloulme & Shaw, 2020). Previous studies have shown that S100A1 protein could bind to RyR receptors to form the S100A1RyR complex, thereby regulating intracellular Ca2+ homeostasis and thus participating in the pathophysiological processes of Alzheimer’s disease (Wright et al., 2008). Another member of the S100 protein family, the S100B protein, shares a similar biological phenotype with the S100A1 protein. The study by Ercole et al. (2016) revealed that the S100B protein could effectively predict the severity of acute cerebral infarction and its prognosis. Given that S100A1 and S100B proteins might function in a common signalling pathway *in vivo* (Ercole et al., 2016; Heizmann, 2019; Afanador et al., 2014), it was acceptable to posit that the S100A1 protein might be utilized in some capacity for disease diagnosis or reaction to the severity of AIS. The S100A1 protein itself was analyzed in terms of inflammation. A study by Yu et al. (2015) found that S100A1 might regulate inflammation and oxidative stress responses in H9C2 cells through the TLR4/NF- *κ*B pathway. Hypoxic cardiomyocytes released endogenous S100A1, which stimulated NF-*κ*B p65 protein by binding to TLR4 receptors, thereby inducing an increase in pro-inflammatory cytokines such as IL-6. In contrast, after eliminating the gene for S100A1 protein by genetic ablation reduced the expression of this protein, a striking finding was observed that intracranial plaque load and number were significantly reduced, as well as the neuroinflammatory response, demonstrating that inhibition of S100A1 protein might be a way to normalize intracranial inflammatory signalling (Afanador et al., 2014). By actively interfering with the S100A1 protein, we discovered that the protein was an indicator of promoting inflammation and exacerbating inflammation, whether from forward or reverse intervention. Therefore, we could think that this protein might be a potential therapeutic indicator. It was rational to speculate that the S100A1 protein might play a role in a similar pathophysiological process in cerebral infarction. To our knowledge, there are no studies on the relevance of S100A1 protein for ischemic cerebral infarction.

In this study, our objective was to determine the level of S100A1 protein in AIS patients early in the disease progression and to test its diagnostic efficacy for the disease. At the same time, the correlation between this protein and the severity of cerebral infarction was judged. Finally, the mechanism of this protein in the pathogenesis of AIS was investigated.

## Materials and Methods

### Study population

This prospective study was conducted at the Brain Center, Department of Neurology, Subei People’s Hospital affiliated to Yangzhou University, China. The study continuously recruited subjects from April 2020 to November 2020. Patients with AIS and TIA were recruited mainly from the hospital’s outpatient and emergency systems, while PEs were mainly collected through the hospital’s Health Screening Center. A total of 192 study subjects were enrolled, including 141 patients with acute ischemic stroke (AIS; age, 67.42  ± 11.35; 39.0% female, 61.0% male), 20 patients with transient ischemic attack (TIA; age, 65.35  ± 7.29; 50.0% female, 50.0% male), and 31 physical examiners (PE, age, 65.97  ± 8.64; 35.9% female, 64.1% male). AIS inclusion criteria were as follows: (1) patients with acute cerebral infarction of the first onset confirmed by cranial CT/MRI who meet the diagnostic criteria of the Chinese Guidelines for the Diagnosis and Treatment of Acute Ischemic Stroke 2018 (Wang et al., 2018; Powers et al., 2018); (2) age greater than 18 years and less than 90 years; (3) obtain informed consent from the patient or family; (4) fasting venous blood samples from the enrolled AIS patients were collected by experienced nurses at a uniform time of 6 a.m. on the day after admission. AIS exclusion criteria were as follows: (1) patients with obvious softening of brain tissue indicated by head CT or MRI; (2) recurrent stroke, or patients with severe infection, severe liver, kidney, heart, and respiratory system diseases, pregnancy, and known malignancies; (3) patients with Alzheimer’s disease or epileptic seizures; (4) patients with rheumatoid arthritis and other autoimmune diseases; (5) patients with various secondary cerebral hemorrhages, intracranial and extracranial injuries, or brain tumour hemorrhages; (6) patients with acute myocardial infarction or previous serious cardiovascular and cerebrovascular adverse events. (7) Patients received intravenous and mechanical thrombolysis in other hospitals or our hospital. TIA inclusion criteria were as follows: (1) meet the diagnostic criteria for TIA published by the American Heart Association/American Stroke Association (Kernan et al., 2014) while the patient was having the first episode (Amarenco, 2020); (2) age greater than 18 years and less than 90 years; (3) obtain informed consent from the patient or family; (4) fasting venous blood samples from the enrolled TIA patients were collected by experienced nurses at a uniform time of 6 a.m. on the day after admission. TIA exclusion criteria were as follows: (1) patients with severe infections, serious liver, kidney, heart, and respiratory system diseases, pregnancy, and known malignancies; (2) patients with Alzheimer’s disease or epileptic seizures; (3) patients with rheumatoid arthritis and other autoimmune diseases; (4) patients with various secondary cerebral hemorrhages, intracranial and extracranial injuries, or various brain tumour hemorrhages; (5) patients with short-term cerebral infarction or significant post-infarction neurological deficits. PE inclusion criteria were as follows: (1) randomly selected subjects who came to the hospital’s Health Screening Center for physical examination and had perfect baseline information; (2) age greater than 18 years and less than 90 years; (3) obtain their informed consent; (4) fasting venous blood samples from the enrolled PEs were collected by experienced nurses at a uniform time of 6 a.m. on the day of physical examination at the Health Screening Center. PE exclusion criteria were as follows: (1) patients with severe infections, serious liver, kidney, heart, and respiratory system diseases, pregnancy, and known malignancies; (2) patients with Alzheimer’s disease or epileptic seizures; (3) patients with rheumatoid arthritis and other autoimmune diseases; (4) patients with various secondary cerebral hemorrhages, intracranial and extracranial injuries, or various brain tumour hemorrhages; (5) patients with short-term cerebral infarction or significant post-infarction neurological deficits. All inclusion and exclusion criteria were completed by two well-trained senior neurologists. This study was approved by the Ethics Committee of Subei People’s Hospital affiliated to Yangzhou University in China, and all the selected patients were signed with informed consent by their legal representatives or close relatives. Its IRB approval number was 2021ky-176.

### Baseline assessments and laboratory measurements

We gathered information about the research participants, such as their demographics, clinical information, risk factors, blood counts, biochemical indicators, and laboratory parameters. Age and sex were demographic traits. Admission NIHSS (range, 0–42, with a higher score suggesting a more severe neurologic impairment), and TOAST type (including large-artery atherosclerosis (LAA), cardioembolism (CE), small-artery occlusion (SAO), and others), admission diastolic blood pressure (BP), and admission systolic BP made up the clinical information. Risk factors included body mass index (BMI), alcohol drinking, smoking, triple-height, and heart disease. Blood counts contained blood glucose (BG), red blood cell (RBC) count, hematocrit count, leukocyte count, neutrophil count, lymphocyte count, monocyte count, red blood cell distribution width (RDW), and platelets (PLT) count. Biochemical indicators included total cholesterol (TC), triglycerides (TG), low-density lipoproteins (LDL), high-density lipoproteins (HDL), creatinine, Alanine transaminase (ALT), aspartate transaminase (AST), and homocysteine. Laboratory parameters contained plasma S100A1 calcium-binding protein, Nuclear factor kappa-B p65 (NF-*κ*B p65), and Interleukin-6 (IL-6). The ELISA kits for these three indicators were purchased from Beijing Green Source Boulder Biotechnology Corporation. The levels of plasma S100A1 protein, NF-*κ*B p65, and IL-6 of the test individuals were assessed by skilled testers in accordance with the ELISA double antibody sandwich method’s usage guidelines. The fasting venous blood samples of the enrolled AIS and TIA patients were collected early on the second day of admission, and the PE group was completed in the hospital’s Health Screening Center. Other blood samples were tested by our experienced inspectors in our Clinical Research Laboratory.

### Calculation and grouping of cerebral infarction volume

The volume of cerebral infarction (according to the Pullicino formula (Liu, 2021)) = the longest diameter × the widest diameter × the number of layers × (layer spacing + layer thickness) × 0.5 shown on head CT/MRI. In the case of multiple cerebral infarctions, the volume of each scattered multiple infarctions was calculated and then added to obtain the total volume. Due to the specificity of the brainstem location, the calculation of infarct volume in this region needs further refinement. Therefore, brainstem infarcts were not included in this study. The main infarct sites in this study were the basal ganglia region, thalamus, occipital lobe, and cerebellar hemispheres. According to the grouping of cerebral infarction volume (Meng & Ji, 2021); small-volume cerebral infarction (SCI) group: total infarct volume <5 cm^3^; middle-volume cerebral infarction (MCI) group: total infarct volume 5–10 cm^3^; large-volume cerebral infarction (LCI) group: total infarct volume >10 cm^3^.

### Statistical analysis

Statistical analysis was completed using Spss.26 software. The Kolmogorov–Smirnov test was used to assess the normal distribution. The mean and standard deviation ($\bar {x}$ ± s)of continuous variables that fit the normal distribution were used as the unit of expression. One-way analysis of variance (ANOVA) was used among the three groups, and the LSD method was used to compare groups. Continuous variables not subject to normal distribution were expressed as the median and interquartile range (25th to 75th percentile); the Kruskal-Wallis H test was used among the three groups, and Bonferroni correction was generally used for comparison between groups. Enumeration data were reported as percentages (%), and when applicable, Fisher’s exact or the *χ*^2^ test were used to compare groups. The independent influencing factors connected to the illness diagnosis were chosen using multivariate logistic regression analysis. The Receiver Operating Characteristic curve (ROC) was used to evaluate the diagnostic efficacy of plasma S100A1 protein, a new inflammatory marker, for AIS and TIA. The area under the curve (AUC), the maximum Youden index (*J*max), the optimal cut-off value (cut-off), the sensitivity (*Se*), and the specificity (*Sp*) were calculated. Pearson or Spearman correlation analysis was used to test the correlation between the levels of plasma S100A1, NF-*κ*B p65 and IL-6 and the volume of cerebral infarction and to explore the correlation among the three. A two-tailed value of *P* < 0.05 was considered significant.

## Results

### Comparison of baseline characteristic data in AIS, TIA, and PE groups

Baseline characteristic data among the AIS, TIA, and PE groups were summarized in Table 1. A total of 192 study subjects were enrolled, including 141 patients with acute ischemic stroke (AIS; age, 67.42 ± 11.35; 39.0% female, 61.0% male), 20 patients with transient ischemic attack (TIA; age, 65.35 ± 7.29; 50.0% female, 50.0% male), and 31 physical examiners (PE, age, 65.97 ± 8.64; 35.9% female, 64.1% male). The three groups had no significant differences in demographic characteristics and risk factors. Admission systolic pressure in the AIS group was significantly higher than that in the TIA group and PE group ((148.24 ± 17.83) *vs* (130.90 ± 4.61) *vs* (130.45 ± 7.15), *P* < 0.001). Comparison between the groups suggested no significant difference between the TIA and PE groups (*P* > 0.05). The test indexes of blood samples include blood routine indexes, biochemical indexes, and laboratory parameters. Leukocytes and creatinine count were significantly increased in the AIS group (Leukocytes, (7.84 ± 5.70) *vs* (5.45 ± 2.01) *vs* (5.41 ± 2.24), *P* = 0.015; Creatinine, (64.10; IQR, 52.85–76.90) *vs* (51.45; IQR, 47.10–75.10) *vs* (54.80; IQR, 43.10–71.10), *P* = 0.009). Comparison between groups only suggested that leukocytes and creatinine count in the AIS group were significantly higher than in the PE group. At the same time, the peripheral blood monocytes, PLT, and ALT were significantly increased in the AIS group (*P* < 0.001). Comparison between groups also indicated that these indexes were significantly higher in the AIS group than in the other two groups (Monocytes, (0.49 ± 0.25) *vs* (0.34 ± 0.13) *vs* (0.33 ± 0.10), *P* < 0.001; PLT, (186.28 ± 59.29) *vs* (146.45 ± 48.99) *vs* (149.58 ± 42.32), *P* < 0.001; ALT, (26.00; IQR, 20.00–38.00) *vs* (34.00; IQR, 34.00–39.75) *vs* (34.00; IQR, 32.00–37.00), *P* < 0.001). BG, RBC, hematocrit, neutrophils, lymphocytes, RDW, TC, TG, LDL, HDL, AST, and homocysteine were similar among the three groups. Laboratory parameters showed statistically significant differences in plasma S100A1, NF-*κ*B P65, and IL-6 among the AIS group, TIA group, and PE group (S100A1, (230.96 ± 39.37) *vs* (185.85 ± 43.24) *vs* (181.47 ± 27.39), *P* < 0.001; NF-*κ*B p65, (3.99 ± 0.65) *vs* (3.58 ± 0.74) *vs* (3.51 ± 0.99), *P* = 0.001; IL-6, (13.32 ± 1.57) *vs* (11.61 ± 1.67) *vs* (11.42 ± 2.34), *P* < 0.001). Comparison between the groups suggested that the S100A1, NF-*κ*B P65, and IL-6 in the AIS group were significantly higher than in the TIA group and PE group (*P* < 0.05). There was no significant difference between the TIA group and PE group (*P* > 0.05).

**Table 1:** Comparison of baseline characteristics data among the AIS, TIA, and PE groups. Baseline characteristic data among the AIS, TIA, and PE groups are summarized in Table 1. Also, whether there was variability between the three groups was compared.

Characteristics	AIS group (*n* = 141)	TIA group (*n* = 20)	PE group (*n* = 31)	*P*-value
Demographics				
Age (years)	67.42 ± 11.35	65.35 ± 7.29	65.97 ± 8.64	0.609
Female (*n*) (%)	55 (39.0)	10 (50.0)	11 (35.5)	0.595
Risk factors				
BMI (kg/m^2^)	24.26 ± 3.18	23.89 ± 3.04	24.86 ± 2.74	0.508
Smoking (*n*) (%)	51 (36.2)	8 (40.0)	9 (29.0)	0.703
Alcohol drinking (*n*) (%)	31 (22.1)	6 (30.0)	8 (25.8)	0.614
Hypertension (*n*) (%)	96 (68.1)	12 (60.0)	19 (61.3)	0.652
Diabetes (*n*) (%)	37 (26.2)	5 (25)	8 (25.8)	0.992
Hyperlipidemia (*n*) (%)	9 (6.4)	1 (5.0)	2 (6.5)	0.971
AF (*n*) (%)	21 (14.9)	2 (10.0)	3 (9.7)	0.694
CHD (*n*) (%)	17 (12.1)	2 (10.0)	3 (9.7)	0.934
Clinical data				
Systolic BP (mmHg)	148.24 ± 17.83	130.90 ± 4.61	130.45 ± 7.15	0.000^*(a,b)^
Diastolic BP (mmHg)	83.10 ± 11.82	81.05 ± 8.09	80.71 ± 9.14	0.461
Blood routine indexes				
BG (mmol/L)	6.31 ± 2.33	5.47 ± 1.07	5.55 ± 1.04	0.066
RBC (10^12^/L)	4.98 ± 0.55	4.46 ± 0.51	4.45 ± 0.49	0.792
Hematocrit (%)	39.67 ± 5.93	38.30 ± 4.20	38.08 ± 4.19	0.250
Leukocytes (10 $\hat {}$9/L)	7.84 ± 5.70	5.45 ± 2.01	5.41 ± 2.24	0.015^*(b)^
Neutrophils (10 $\hat {}$9/L)	4.79 ± 2.18	4.32 ± 1.31	4.14 ± 1.49	0.203
Lymphocytes (10 $\hat {}$9/L)	1.50 ± 0.65	1.51 ± 0.28	1.58 ± 0.27	0.785
Monocytes (10 $\hat {}$9/L)	0.49 ± 0.25	0.34 ± 0.13	0.33 ± 0.10	0.000^*(a,b)^
RDW (%)	12.80 ± 1.39	12.46 ± 0.97	12.25 ± 0.97	0.076
PLT (10 $\hat {}$9/L)	186.28 ± 59.29	146.45 ± 48.99	149.58 ± 42.32	0.000^*(a,b)^
Biochemical indicators				
TC (mmol/L)	4.10 ± 0.87	3.91 ± 0.68	3.98 ± 0.56	0.507
TG (mmol/L)	1.66 ± 0.82	1.56 ± 0.17	1.53 ± 0.36	0.599
LDL (mmol/L)	2.48 ± 0.74	2.34 ± 0.49	2.40 ± 0.45	0.662
HDL (mmol/L)	1.01 ± 0.29	1.03 ± 0.20	1.01 ± 0.21	0.969
Creatinine (umoL/L)	64.10 (52.85,76.90)	51.45 (47.10,75.10)	54.80 (43.10,71.10)	0.009^*(b)^
ALT (u/L)	26.00 (20.00,38.00)	34.00 (34.00,39.75)	34.00 (32.00,37.00)	0.000^*(a,b)^
AST(u/l)	26.00 (21.00,32.5)	24.50 (22.00,35.50)	27.00 (20.00,33.00)	0.988
Homocysteine (umol/L)	10.00 (6.00,13.50)	8.50 (7.2515.00)	9.00 (6.00,14.00)	0.942
Laboratory parameters				
S100A1 (pg/ml)	230.96 ± 39.37	140.85 ± 43.24	136.47 ± 27.39	0.000^*(a,b)^
NF-*κ*B p65 (ng/ml)	3.99 ± 0.65	2.88 ± 0.74	2.81 ± 0.99	0.001^*(a,b)^
IL-6 (pg/ml)	13.32 ± 1.57	9.61 ± 1.67	9.42 ± 2.34	0.000^*(a,b)^

**Notes.**

Abbreviations AIS Acute ischemic stroke TIA Transient ischemic attack PE physical examiners BMI Body mass index AF Atrial fibrillation CHD Coronary heart disease BP Blood pressure BG Blood glucose RBC Red blood cell RDW Red blood cell distribution width PLT Platelet TC Total cholesterol TG Triglycerides LDL Low-density lipoproteins HDL High-density lipoproteins ALT Alanine transaminase AST Aspartate transaminase NF- *κ*B p65 Nuclear factor kappa-B p65 IL-6 Interleukin-6

*, statistically significant (*P* < 0.05) between the three groups; a, statistically significant (*P* < 0.05) between the AIS and TIA groups; b, statistically significant (*P* < 0.05) between the AIS and PE groups; c, statistically significant (*P* < 0.05) between the TIA and PE groups.

### Multivariate logistic regression analysis for the diagnosis of disease

The type of disease was taken as the dependent variable, and systolic BP, leukocytes, monocytes, PLT, creatinine, ALT, S100A1, NF-*κ*B p65, and IL-6 were taken as independent variables. Multivariate logistic regression analysis showed that the values of systolic BP, ALT, and S100A1 were correlated with the diagnosis of disease, and their Chi-square values were 28.365 (Df, 2; *P* < 0.001), 14.308 (Df, 2; *P* = 0.001), 26.036 (Df, 2; *P* < 0.001). The level of plasma S100A1 might be an independent predictive factor for disease diagnosis. Systolic BP and ALT might also be autogenous predictors of the diagnosis of different types of disease (Table 2).

**Table 2:** Multivariate logistic regression analysis for the diagnosis of disease. The type of disease was taken as the dependent variable, and systolic BP, leukocytes, monocytes, PLT, creatinine, ALT, S100A1, NF-*κ*B p65, and IL-6 were taken as independent variables. Multivariate logistic regression analysis revealed significant variables.

	−2 log-likelihood of reduced model	Chi-square	Df	*P*-value
Intercept	225.716	67.677	2	0.000^*^
Systolic BP	186.403	28.365	2	0.000^*^
Leukocytes	163.726	5.687	2	0.058
Monocytes	166.904	8.865	2	0.012
PLT	159.892	1.854	2	0.396
Creatinine	159.072	1.033	2	0.597
ALT	172.346	14.308	2	0.001^*^
S100A1	184.074	26.036	2	0.000^*^
NF-*κ*B p65	158.169	0.130	2	0.937
IL-6	159.015	0.977	2	0.614

**Notes.**

Abbreviations Df Degrees of freedom BP Blood pressure PLT Platelet ALT Alanine transaminase NF- *κ*B p65 Nuclear factor kappa-B p65 IL-6 Interleukin-6

* This factor was statistically significant for disease diagnosis (*P* < 0.05).

### Diagnostic efficacy of plasma S100A1 protein for AIS and TIA

**Figure 1:**
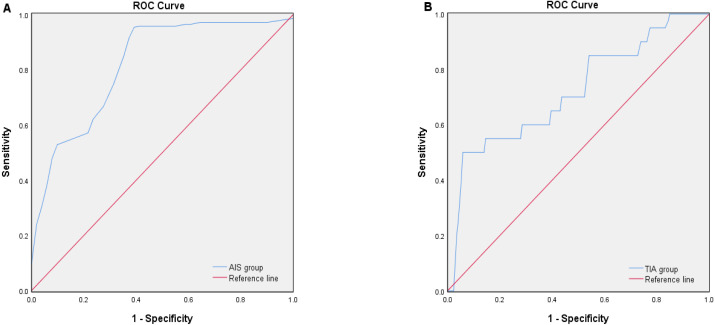
ROC curve analysis of the diagnostic efficacy of plasma S100A1 protein on AIS (A) and TIA (B). The area below the blue curve represents the diagnostic efficiency of the disease. The larger the area, the stronger the diagnostic efficiency.

The diagnostic efficacy of AIS and TIA was assessed using the receiver operating characteristic curve (ROC) (Fig. 1). The area under the ROC curve (AUC) of plasma S100A1 for the diagnosis of AIS was 0.818 (Fig. 1A) (*P* < 0.001, 95% confidence interval [CI] 0.749–0.887), when the optimum cutoff value (cut off) was 181.03, its maximum Youden index (*J*max) was 0.578, sensitivity (*Se*) was 95.0%, and specificity (*Sp*) was 62.7% (Table 3). The AUC of plasma S100A1 for the diagnosis of TIA was 0.720 (Fig. 1B) (*P* = 0.001, 95% CI [0.592–0.848]), and when cut off was 150.14, its *J*max was 0.442, *Se* was 50.0%, and *Sp* was 94.2% (Table 3).

### Comparison of baseline characteristics between groups with different cerebral infarction volumes

**Figure 2:**
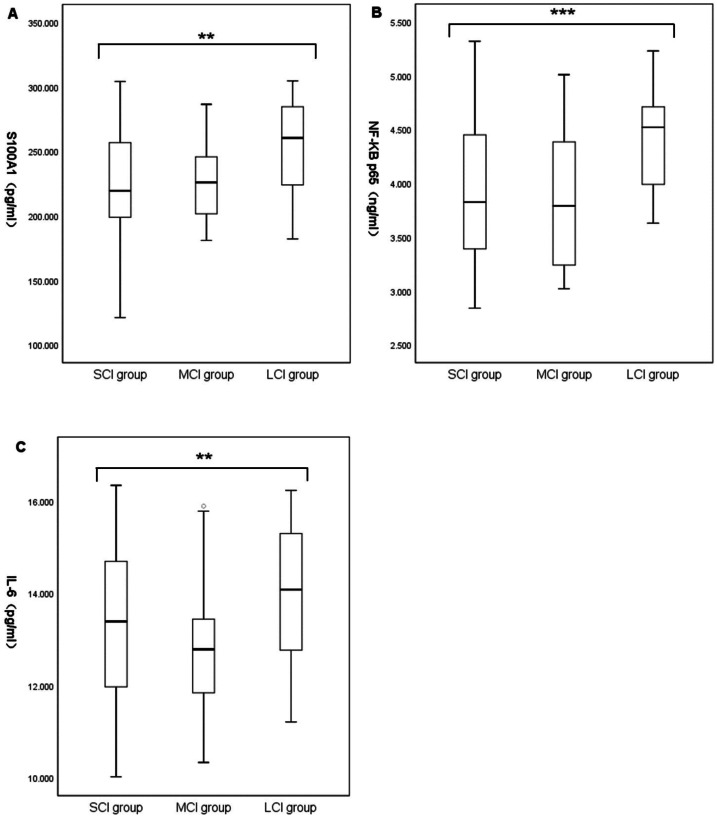
Comparison of plasma S100A1 protein (A), NF-*κ*B p65 (B), and IL-6 (C) among patients with different cerebral infarction volumes. The differences of plasma S100A1 protein, NF-*κ*B P65 and IL-6 in large, medium and small volume cerebral infarction groups were compared. Two asterisks (**) indicate statistically significant differences among the three groups at the 0.01 level; three asterisks (***) indicate statistically significant differences among the three groups at the 0.001 level.

Baseline characteristic data for the different cerebral infarction volume groups were summarized in Table 4. A total of 141 patients with acute ischemic stroke were enrolled, including 78 in the small-volume cerebral infarction group (SCI; age, 67.65 ± 10.28; 34.6% female, 65.4% male), 32 in the medium-volume cerebral infarction group (MCI; age, 66.50 ± 13.41; 44.1% female, 55.9% male), and 31 in the large-volume cerebral infarction group (LCI; age, 67.77 ± 11.97; 41.9% female, 58.1% male). There were no significant differences in demographic characteristics, blood routine indexes, and risk factors among the three groups. Admission NIHSS in the LCI group was significantly higher than in the MCI group and SCI group ((11.00; IQR, 4.00–22.00) *vs* (2.50; IQR, 1.25–4.75) *vs* (2.00; IQR, 1.00–3.25), *P* < 0.001). Comparison between the groups suggested no significant difference between the SCI and MCI groups (*P* > 0.05). ALT and AST count were significantly increased in the LCI group (ALT, (36.00; IQR, 25.00–49.00) *vs* (24.00; IQR, 19.25–38.00) *vs* (24.00; IQR, 17.75–34.25), *P* = 0.002; AST, (29.00; IQR, 25.00–40.00) *vs* (25.50; IQR, 22.00–29.75) *vs* (25.00; IQR, 19.75–30.25), *P* = 0.007). Comparison between groups only suggested that ALT and AST counts in the LCI group were significantly higher than in the SCI group. Systolic BP, diastolic BP, TOAST type, TC, TG, LDL, HDL, creatinine, and homocysteine were similar among the three groups. Laboratory parameters showed statistically significant differences in plasma S100A1, NF-*κ*B P65, and IL-6 among the three groups. Comparison between the groups suggested that the levels of S100A1, NF-*κ*B P65, and IL-6 in the LCI group were significantly higher than those in the MCI and SCI group (S100A1, (254.25 ± 37.07) *vs* (225.42 ± 30.92) *vs* (223.98 ± 40.21), *P* = 0.001; NF-*κ*B p65, (4.41 ± 0.45) *vs* (3.85 ± 0.64) *vs* (3.88 ± 0.66), *P* < 0.001; IL-6, (14.00 ± 1.40) *vs* (12.77 ± 1.31) *vs* (13.27 ± 1.65), *P* = 0.007) (Fig. 2). There was no significant difference between the MCI and SCI groups (*P* > 0.05).

**Table 3:** Diagnostic efficacy of plasma S100A1 protein for AIS and TIA.

	AUC	95% CI	*P*-value	*J*_max_	Cut-off	*Se* (%)	*Sp* (%)
AIS group	0.818	0.749–0.887	0.000^*^	0.578	181.03	95.0	62.7
TIA group	0.720	0.592–0.848	0.001^*^	0.442	150.14	50.0	94.2

**Notes.**

* S100A1 protein was statistically significant for disease diagnosis (*P* < 0.05). The higher the value of AUC, the stronger the diagnostic effect.

**Table 4:** Comparison of baseline characteristics between groups with different cerebral infarction volumes. Baseline characteristic data for the different cerebral infarction volume groups are summarized in Table 4. At the same time, whether there was variability between the three groups was compared.

	SCI group (*n* = 78)	MCI group (*n* = 32)	LCI group (*n* = 31)	*P*-value
Demographics				
Age (years)	67.65 ± 10.28	66.50 ± 13.41	67.77 ± 11.97	0.874
Female (*n*) (%)	27 (34.6)	15 (44.1)	13 (41.9)	0.455
Risk factors				
BMI (kg/m^2^)	24.01 ± 3.19	24.09 ± 2.82	25.07 ± 3.47	0.278
Smoking (*n*) (%)	30 (38.5)	10 (31.3)	11 (35.5)	0.771
Alcohol drinking (*n*) (%)	17 (21.8)	6 (18.8)	8 (25.8)	0.793
Hypertension (*n*) (%)	53 (67.9)	22 (68.8)	21 (67.7)	0.343
Diabetes (*n*) (%)	22 (28.2)	8 (25.0)	7 (22.6)	0.844
Hyperlipidemia (*n*) (%)	4 (5.1)	3 (9.4)	2 (6.5)	0.730
AF (*n*) (%)	11 (14.1)	6 (18.8)	4 (12.9)	0.810
CHD (*n*) (%)	9 (11.5)	4 (12.5)	4 (12.9)	1.000
Clinical data				
Systolic BP(mmHg)	82.96 ± 11.70	84.09 ± 13.57	82.42 ± 10.45	0.846
Diastolic BP(mmHg)	150.09 ± 18.45	147.66 ± 19.68	144.19 ± 13.54	0.293
Admission NIHSS (points)	2.00 (1.00,3.25)	2.50 (1.25,4.75)	11.00 (4.00,22.00)	0.000^*(b,c)^
TOAST type				
LAA (*n*) (%)	30 (38.5)	12 (37.5)	11 (35.5)	0.972
CE (*n*) (%)	11 (14.1)	6 (18.8)	4 (12.9)	0.810
SAO (*n*) (%)	35 (44.9)	14 (43.8)	15 (48.4)	0.947
Others (*n*) (%)	2 (2.6)	0 (0.0)	1 (3.2)	0.790
Blood routine indexes				
BG (mmol/L)	6.00 ± 2.39	2.39 ± 2.60	6.70 ± 1.78	0.212
RBC (10^12^/L)	5.36 ± 7.37	4.44 ± 0.67	4.58 ± 0.63	0.659
Hematocrit (%)	40.17 ± 6.67	39.09 ± 4.38	39.03 ± 5.34	0.547
Leukocytes (10 $\hat {}$9/L)	7.91 ± 6.23	7.77 ± 6.59	7.72 ± 2.64	0.986
Neutrophils (10 $\hat {}$9/L)	4.63 ± 2.17	4.45 ± 1.42	5.56 ± 2.70	0.080
Lymphocytes (10 $\hat {}$9/L)	1.55 ± 0.73	1.52 ± 0.52	1.35 ± 0.52	0.358
Monocytes (10 $\hat {}$9/L)	0.48 ± 0.26	0.52 ± 0.28	0.48 ± 0.19	0.749
RDW (%)	12.82 ± 1.50	12.79 ± 1.26	12.73 ± 1.28	0.950
PLT (10 $\hat {}$9/L)	191.81 ± 59.92	179.81 ± 55.87	179.06 ± 61.51	0.471
Biochemical indicators				
TC (mmol/L)	4.07 ± 0.85	4.08 ± 0.82	4.20 ± 1.00	0.774
TG (mmol/L)	1.65 ± 0.71	1.84 ± 0.97	1.49 ± 0.89	0.233
LDL (mmol/L)	2.48 ± 0.74	2.36 ± 0.71	2.58 ± 0.78	0.480
HDL (mmol/L)	0.99 ± 0.24	0.98 ± 0.27	1.11 ± 0.38	0.115
Creatinine (umoL/L)	63.80 (52.78,76.23)	64.4 (47.45,78.38)	64.50 (57.90,89.30)	0.448
ALT (u/L)	24.00 (17.75,34.25)	24.00 (19.25,38.00)	36.00 (25.00,49.00)	0.002^*(b)^
AST(u/l)	25.00 (19.75,30.25)	25.50 (22.00,29.75)	29.00 (25.00,40.00)	0.007^*(b)^
Homocysteine (umol/L)	9.50 (5.00,14.00)	10.00 (7.00,14.75)	9.00 (8.00,12.00)	0.858
Laboratory parameters				
S100A1 (pg/ml)	223.98 ± 40.21	225.42 ± 30.92	254.25 ± 37.07	0.001^*(b,c)^
NF-*κ*B p65 (ng/ml)	3.88 ± 0.66	3.85 ± 0.64	4.41 ± 0.45	0.000^*(b,c)^
IL-6 (pg/ml)	13.27 ± 1.65	12.77 ± 1.31	14.00 ± 1.40	0.007^*(b,c)^

**Notes.**

Abbreviations SCI Small-volume cerebral infarction MCI Middle-volume cerebral infarction LCI Large-volume cerebral infarction BMI Body mass index AF Atrial fibrillation CHD Coronary heart disease BP Blood pressure NIHSS National Institutes of Health Stroke Scale TOAST Trial of Org 10172 in acute stroke treatment LAA Large-artery atherosclerosis CE Cardioembolism SAO Small-artery occlusion BG Blood glucose RBC Red blood cell RDW Red blood cell distribution width PLT Platelet TC Total cholesterol TG Triglycerides LDL Low-density lipoproteins HDL High-density lipoproteins ALT Alanine transaminase AST Aspartate transaminase NF- *κ*B p65 Nuclear factor kappa-B p65 IL-6 Interleukin-6

*, statistically significant (*P* < 0.05) between the three groups; a, statistically significant (*P* < 0.05) between the SCI and MCI groups; b, statistically significant (*P* < 0.05) between the SCI and LCI groups; c, statistically significant (*P* < 0.05) between the MCI and LCI groups.

### Correlation of cerebral infarct volume with the levels of S100A1 protein, NF-*κ*B p65, and IL-6

**Figure 3:**
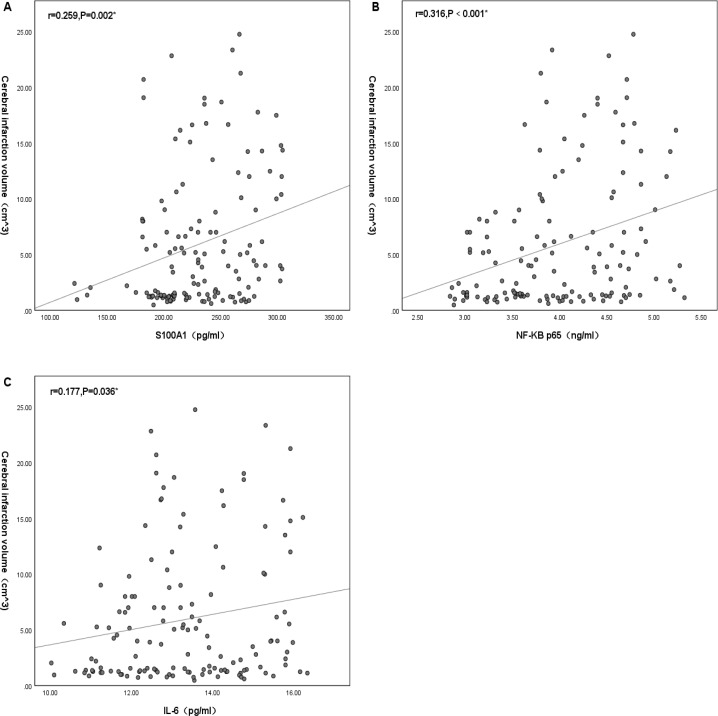
Correlation of cerebral infarction volume with the levels of S100A1 protein (A), NF-*κ*B p65 (B), and IL-6 (C). An asterisk (*) indicates that this factor was statistically associated with cerebral infarction volume (*P* < 0.05). The greater the correlation coefficient, the stronger the correlation.

Plasma S100A1, NF-*κ*B P65, and IL-6 significantly differed from cerebral infarction volume. There was no significant positive correlation between plasma S100A1 and cerebral infarction volume (*R* < 0.3), but there was statistical significance (*r* = 0.259, *P* = 0.002) (Fig. 3A). There was a positive correlation between NF-*κ*B p65 and cerebral infarction volume with statistical significance (*r* = 0.316, *P* < 0.001) (Fig. 3B). There was no significant positive correlation between IL-6 and cerebral infarction volume (*R* < 0.3), but there was statistical significance (*r* = 0.177, *P* = 0.036) (Fig. 3C).

### Mechanistic investigation: correlation analysis among the levels of plasma S100A1 protein, NF-*κ*B p65, and IL-6

**Figure 4:**
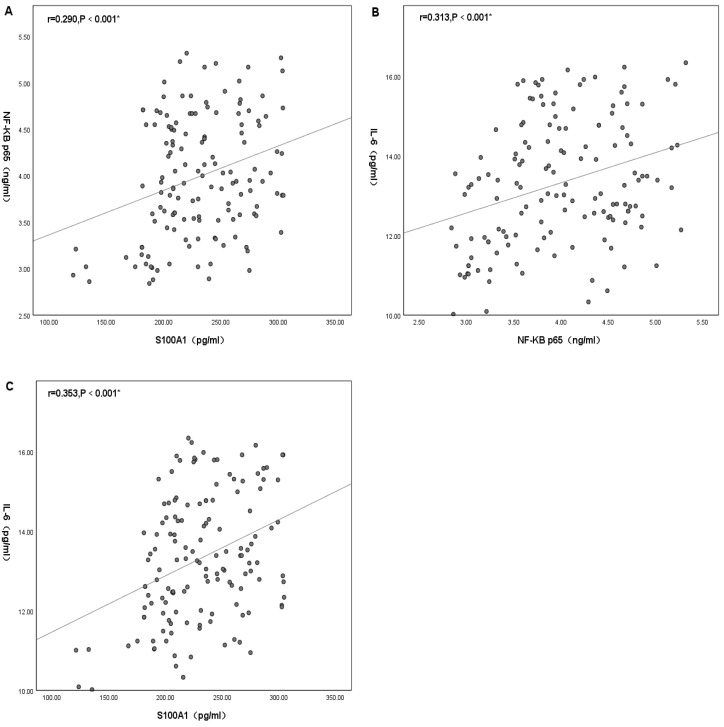
Mechanistic investigation: correlation analysis among the levels of plasma S100A1 protein (A), NF-*κ*B p65 (B), and IL-6 (C). An asterisk (*) indicates that there was a statistically significant correlation between the two-factor levels (*P* < 0.05). The greater the correlation coefficient, the stronger the correlation.

Statistically significant differences were found between plasma S100A1, NF-*κ*B P65 and IL-6. There was no significant positive correlation between plasma S100A1 and NF-*κ*B p65 (*R* < 0.3), but there was statistical significance (*R* = 0.290, *P* < 0.001) (Fig. 4A). There was a positive correlation between plasma IL-6 and NF-*κ*B P65 with statistical significance (*R* = 0.313, *P* < 0.001) (Fig. 4B). There was a positive correlation between plasma S100A1 and IL-6 with statistical significance (*R* = 0.353, *P* < 0.001) (Fig. 4C).

## Discussion

In this study, we first assessed the diagnostic efficacy of plasma S100A1 protein for AIS and TIA diseases. The results showed that the diagnosis of AIS might be more favorable when S100A1 ≥181.03 pg/ml, while the diagnosis of TIA might be more advantageous when 150.14 pg/ml ≤S100A1 ≤181.03 pg/ml. At the same time, the correlation between plasma S100A1 protein and cerebral infarct volume was proposed in this study, and the results showed that its level could reflect the severity of AIS to some extent. Furthermore, this study also found that plasma S100A1 protein levels and IL-6 and NF-*κ*B p65 correlated with each other. It was reasonably speculated that this protein might mediate inflammatory responses through the NF-*κ*B pathway in the pathophysiological process of AIS.

Inflammation, abnormal coagulation, and atherosclerosis all play important roles in various pathophysiological stages of AIS (Atif et al., 2020; Fani et al., 2020; Li, Qiu & Li, 2021). Both in the development process of AIS and the post-AIS process, it has a crucial significance. S100A1 protein was a novel inflammatory marker and a protein subtype in the S100 family of proteins (Baudier, Deloulme & Shaw, 2020). This protein could usually bind calcium ions and was essentially a type of calcium-binding protein traditionally found only in vertebrates (Chaturvedi et al., 2020). Currently, there are 25 known members of the S100 protein family with a high degree of sequence and structural similarity. They are involved in intracellular and extracellular regulatory activities in various tissues. In the nervous system, intracellular and extracellularly, S100 proteins could stimulate neuronal and glial cell proliferation and cause neuronal death through apoptotic programs, stimulating or inhibiting cellular inflammation (Sreejit et al., 2020). In recent years, S100A1 protein has been well studied in various systemic diseases, including acute myocardial infarction (Fan et al., 2019), heart failure (Soltani, Kheirouri & Enamzadeh, 2021), malignancy (Nakagawa et al., 2022; Li et al., 2021; Wang et al., 2021b), *etc*. Previous studies have shown that hypoxic cardiomyocytes released endogenous S100A1, which stimulated NF- *κ*B p65 by binding to TLR4, thereby inducing an increase in pro-inflammatory cytokines; either endogenous or exogenous S100A1 significantly enhanced NF-*κ*B phosphorylation in cardiomyocytes, thereby activating the expression of multiple pro-inflammatory cytokines involved in the inflammatory response (*e.g.*, tumour necrosis factor *α*, chemokines, and interleukin 6) (Yu et al., 2015). This was the theoretical basis of the present study. Therefore, we suspected whether there was also an association between plasma S100A1 protein levels and AIS and the relationship with NF-*κ*B p65 and IL-6 levels downstream of the conduction pathway.

In this study, clinical data were first compared among the AIS, TIA, and PE groups, and statistically significant differences were found in plasma S100A1, NF-*κ*B p65, and IL-6 (*P* < 0.05). A two-by-two comparison revealed that the levels of the three in the AIS group were significantly higher than in the TIA and PE groups (*P* < 0.05). At the same time, no statistically significant differences were discovered between the TIA and PE groups. The results were more similar to the findings of Fan et al. (2019) in acute myocardial infarction, which indicated that plasma S100A1 levels were significantly higher in the ST-segment elevation myocardial infarction (STEMI) group than in healthy controls. After multifactorial logistic regression analysis, it was shown that S100A1 was an independent predictor of STEMI and that patients who possessed higher levels of S100A1 protein had a significantly increased risk of STEMI. It was finally concluded that the elevated plasma S100A1 concentration was an essential predictor of STEMI. This was generally consistent with the finding that the S100A1 level in the AIS group was significantly higher than that in the PE group in this study. This study also found that the S100A1 level in the AIS group was also higher than in the TIA group. It was presumed that this might be associated with the different severity of inflammation between the patients in the AIS and TIA groups, where the clinical symptoms of the patients in the TIA group could be rapidly relieved in a short period, and their resulting inflammatory response was relatively mild. Previous studies have shown that transient ischemic symptoms usually last for seconds or minutes and typically last less than an hour (Amarenco, 2020). The new definition of TIA is now “tissue-based”. An ischemic lesion is not visible on brain imaging in a patient with TIA, and a patient with transient symptoms who has even a tiny ischemic brain lesion on imaging is considered to have had a minor ischemic stroke (Amarenco, 2020; Albers et al., 2002; Kleindorfer et al., 2021). Similarly, in the study by Wang et al. (2021a), the overall level of inflammation in TIA patients was generally lower, which might be relevant to the short duration of TIA symptoms. Hence, both from an inflammatory and imaging point of view, TIA patients tended to be more normal in their performance. This nicely explained the insignificant differences in this study’s three detection indexes between the TIA and PE groups. In addition, multivariate logistic regression analysis showed that plasma S100A1 protein might be an independent predictor of disease diagnosis, which was also consistent with the conclusion of Fan et al. (2019). From the diagnostic efficacy of S100A1 protein for AIS and TIA, the diagnostic efficacy of S100A1 protein for AIS was higher than that for TIA (AUC of AIS was 0.818 > AUC of TIA was 0.720). According to the performance of the best Cut-off value, the diagnosis of AIS might be more inclined when S100A1 ≥181.03 pg/ml, while the diagnosis of TIA might be inclined when 150.14 pg/ml ≤S100A1 ≤181.03 pg/ml, and more inclined to normal when S100A1 ≤150.14 pg/ml. Thus, depending on the magnitude of this value, it was then feasible to make a more accurate and standardized diagnosis or differential diagnosis of the disease as early as possible in the development of the disease.

In exploring the differences in plasma S100A1 levels in acute ischemic strokes of different severity, it was found that there was variability in S100A1 levels in patients with different volumes of cerebral infarction. The S100A1 levels in the LCI group were significantly higher than those in the MCI and SCI groups (*P* < 0.05). At the same time, no statistically significant difference was found between the MCI and SCI groups (*P* > 0.05). The levels of NF-*κ*B p65 and IL-6 showed similar consequences among the three groups. Plasma S100A1 protein, NF-B p65, and IL-6 were well-established markers for the overall inflammatory status of the patient’s body, according to several earlier research (Rohde et al., 2014; Mizuno et al., 2022; Liu, Chen & Chang, 2022; García-Juárez & Camacho-Morales, 2022). Thus, patients in the LCI group had significantly higher levels of inflammation than those in the MCI and SCI groups. This suggested that larger infarcts might represent higher levels of inflammation. This was similar to the results of Liu, Xiang & Jin (2015) and Wang et al. (2019), overexpression of inflammatory factors in acute cerebral infarction might increase the volume and edema degree of cerebral infarction. Similarly, serum concentrations of inflammatory factor C1q were positively correlated with both infarct volume and NIHSS in patients with AIS in a research by Wang et al. (2020). As was already noted, the size of the infarct determined how much damage to the brain tissue might occur as well as how intensely the inflammatory reaction would be. This study also investigated the relationship between plasma S100A1 and cerebral infarct size, and there was a statistically significant difference between the two (*r* = 0.259, *P* < 0.05), but the correlation coefficient was low. This might be due to the fact that plasma S100A1 protein was not released from brain tissue alone. Still, other systemic and local inflammatory responses, including the effects of certain underlying diseases, might also lead to changes in plasma S100A1 levels. Even nevertheless, plasma S100A1 levels in the LCI group were significantly higher than in the MCI and SCI groups with statistically significant differences, suggesting that S100A1 levels might in part reflect the severity of patients at the time of admission during the acute exacerbation. Meanwhile, the present study also compared the relationship between NF-*κ*B p65, IL-6, and cerebral infarct volume, and the results also showed statistically significant but relatively low correlation coefficients. Previous studies have shown a moderate to a high positive correlation between these two factors and cerebral infarct volume (Li & Xin, 2021; Purroy et al., 2021). The limited sample size of the research population may have contributed to the fact that the same conclusion was not achieved in this work. The levels of plasma S100A1, NF-*κ*B p65, and IL-6 were not statistically significantly different between the MCI and SCI groups, and it was conjectured that these inflammatory markers might need to reach a specific volume of cerebral infarction before their differences could be revealed, but the specific boundaries needed to be further investigated.

Additionally, in this study, the correlation between plasma S100A1, NF-*κ*B p65 and IL-6 was also compared. It turned out that there was some correlation between these three. All three were statistically significantly different from each other, with correlation coefficients preserved at around 0.3, showing a low correlation. This might be due to the complex pathophysiological mechanisms of cerebral infarction and the small number of studied samples. As previously indicated, S100A1 might regulate the inflammatory response of H9C2 cells through the TLR4 /NF-*κ*B pathway. Necrosis of hypoxic cardiac tissue released endogenous S100A1 to activate TLR4, which in turn induced IL-6 production by binding to TLR4 and thus stimulating NF-*κ*B p65 (Yu et al., 2015). This same perspective was shown in the research of Rohde et al. (2014). In studies of multiple sclerosis (MS), it was found that the S100A protein similarly induced microglia activity by activating the NF- *κ*B pathway, thereby increasing the expression of inflammatory cytokines (Wu et al., 2018). Along the same lines, in monocytes with primary thrombocytopenia (PT), IL6 decreased NF-*κ*B-mediated expression of S100A protein levels through a negative feedback mechanism in PT (Diklić et al., 2020). In other words, S100A could enhance IL-6 expression by inducing the NF-*κ*B pathway. In summary, it could be hypothesized that the elevated levels of S100A1 in patients with acute cerebral infarction might enhance the expression of the inflammatory factor IL-6 and thus mediated the inflammatory response through the NF-*κ*B pathway, which was also consistent with the conclusions obtained in this paper.

There were several limitations to this report. First of all, an appropriate sample size and single-center study were important methodological defects, which required an expanded sample size and multi-center prospective study in the later period. Second, the time point at which plasma S100A1 protein levels were determined was single, which could not indicate the dynamic changes in this protein’s levels. Therefore, this protein’s dynamic monitoring and long-term follow-up should be strengthened in the future. Third, further grouping tests have to be conducted since it was possible that the experimental results would be impacted by including patients with anterior and posterior circulation cerebral infarction in the research sample. Fourth, whether plasma S100A1 protein actually enhanced the expression of the inflammatory factor IL-6 through the NF-*κ*B pathway in patients with acute cerebral infarction remained further confirmed by animal experiments. Finally, the possibility that other unknown confounders might influence the results could not be completely ruled out.

## Conclusions

In summary, our study indicated that the plasma S100A1 protein was significantly correlated with the diagnostic value of AIS and the volume of cerebral infarction. This protein might have a better diagnostic effect for AIS and TIA. The diagnosis of AIS might be more favorable when S100A1 ≥181.03 pg/ml, while the diagnosis of TIA might be more advantageous when 150.14 pg/ml ≤S100A1 ≤181.03 pg/ml. Furthermore, plasma S100A1 protein was associated with infarct volume in AIS, and its level reflected the severity of acute cerebral infarction to a certain extent. Increased levels of this protein might mean more severe damage in AIS patients with larger brain infarct volumes, while patients with lower levels could benefit more. Concurrently, there was a correlation between plasma S100A1 protein levels and IL-6 and NF-*κ*B p65, and it was reasonable to speculate that this protein might mediate the inflammatory response through the NF-*κ*B pathway during the pathophysiology of acute cerebral infarction. Based on our findings and previous studies, plasma S100A1 protein has the potential to serve as an inclusion criterion for future clinical diagnostic trials in AIS, and lowering the level of this protein may be a novel therapeutic target to improve their severity.

##  Supplemental Information

10.7717/peerj.14440/supp-1 Supplemental Information 1 Raw data on AIS, TIA and PE groups and between different cerebral infarct volume groups

## Additional Information and Declarations

### Data Availability

The following information was supplied regarding data availability: The raw data is available in the Supplemental File.
